# *Bothriocestus claviceps* (Cestoda: Bothriocephalidae) in pike in southwestern France (Teleostei: Esocidae): molecular evidence

**DOI:** 10.1051/parasite/2026046

**Published:** 2026-08-26

**Authors:** Laura Jamet, Vincent Haÿ, Mélyne Hautecœur, Marion Escarpit, Vincent Renard, Clarice Salaun, Louis Guyon, Agathe Lindemann, Aude Jabbari, Aurélia L’Hostis, Gaël P.J. Denys, Fabienne Audebert

**Affiliations:** 1 UMR Biologie des Organismes et Écosystèmes Aquatiques (BOREA 8067), Sorbonne Université, MNHN, CNRS, IRD, UA 57 rue Cuvier CP26 75005 Paris France; 2 UMR Bioarchéologie, Interactions Sociétés Environnement (BioArch 7209), MNHN, CNRS 43 rue Buffon, CP56 75005 Paris France; 3 Unité Patrimoine Naturel – Centre d’expertise et de données (2006) OFB, MNHN, CNRS, IRD, Muséum national d’Histoire naturelle 36 rue Geoffroy-Saint-Hilaire CP 41 75005 Paris France; 4 Fédération Départementale des Landes pour la Pêche et la Protection des Milieux Aquatiques 102 allées Marines 40400 Tartas France; 5 Union des Fédérations pour la pêche et la protection du milieu aquatique du Bassin Adour-Garonne 5 chemin de Bramofan 31120 Roques France

**Keywords:** *Esox aquitanicus*, Parasitic flatworm, Freshwater, Molecular identification, COI, 28S

## Abstract

*Bothriocestus claviceps* (Goeze, 1782) is a cestode parasite infecting several fish species. One of its principal definitive hosts is the European eel *Anguilla anguilla*. The parasite is widely distributed across Europe and has been reported from numerous countries, such as Poland, Czechia, and France. In southern France, the European eel co-occurs with the Aquitanian pike, *Esox aquitanicus*, an endemic species. This pike is threatened by human activities and hybridization with the introduced Northern pike *Esox lucius* and is therefore classified as “vulnerable” on the French Red List of the International Union for Conservation of Nature (IUCN). Despite its conservation status, little information is available regarding its biology, particularly its parasitofauna, which remains undocumented. Over a three-year period, Aquitanian pike were collected and examined for parasites. Cestodes recovered from the intestinal tract were analyzed using a molecular approach. Molecular identification of the species was based on partial sequences of 28S ribosomal DNA and cytochrome oxidase subunit I (COI). Morphometric data were also obtained to provide additional data on the identified cestode species. Our results provide the first evidence of the presence of *B. claviceps* in the intestine of *E. aquitanicus,* as well as in one hybrid (*E. aquitanicus* × *E. lucius),* and suggest that these fish can act as definitive or post-cyclic hosts for this cestode species. This study contributes new insights into the parasitofauna of the Aquitanian pike and expands current knowledge of *B. claviceps.*

## Abstract (fr)

*Bothriocestus claviceps* (Goeze, 1782) est un cestode parasite infectant plusieurs espèces de poissons. L’un de ses principaux hôtes définitifs est l’anguille européenne, *Anguilla anguilla*. Ce parasite est largement répandu en Europe et a été signalé dans de nombreux pays, tels que la Pologne, la République tchèque et la France. Dans le sud de la France, l’anguille européenne cohabite avec le brochet aquitain, *Esox aquitanicus*, une espèce endémique. Ce brochet est menacé par les activités humaines et l’hybridation avec le brochet du Nord (*Esox lucius*), une espèce introduite ; il est par conséquent classé comme « vulnérable » sur la Liste rouge française de l’Union internationale pour la conservation de la nature (UICN). Malgré son statut de conservation, peu d’informations sont disponibles sur sa biologie, et notamment sur sa parasitofaune, qui reste méconnue. Sur une période de trois ans, des brochets aquitains ont été collectés et examinés à la recherche de parasites. Les cestodes récoltés dans le tractus intestinal ont été analysés par une approche moléculaire. L’identification moléculaire de l’espèce s’est fondée sur des séquences partielles de l’ADN ribosomal 28S et de la sous-unité I de la cytochrome oxydase (COI). Des données morphométriques ont également été recueillies afin d’apporter des informations complémentaires sur l’espèce de cestode identifiée. Nos résultats apportent la première preuve de la présence de *B. claviceps* dans l’intestin de *E. aquitanicus*, ainsi que chez un hybride (*E. aquitanicus* × *E. lucius*), et suggèrent que ces poissons peuvent servir d’hôtes définitifs ou d’hôtes post-cycliques pour cette espèce de cestode. Cette étude apporte de nouvelles connaissances sur la parasitofaune du brochet aquitain et enrichit les données actuelles concernant *B. claviceps*.

## Introduction

The Aquitanian pike *Esox aquitanicus* Denys, Dettai, Persat, Hautecœur & Keith, 2014 (Teleostei: Esociformes) is an endemic species of southwestern France, from the Charente drainage south to the Adour drainage [17]. Compared with other European freshwater fish species, knowledge regarding this pike species remains particularly limited [45], making the development of effective conservation and management strategies difficult. *Esox aquitanicus* lives in small tributaries or coastal catchments and lagoons and these poor environments (sandy substrates, little aquatic vegetation, low biomass) impact its growth [50]. This pike shares its habitats with other endemic fishes of the Garonne and Adour catchments, including sculpins (*Cottus* spp.), gudgeons (*Gobio* spp.), minnows (*Phoxinus* spp.), daces (*Leuciscus* spp.), and stone loaches (*Barbatula* spp.), as well as with diadromous fishes such as the European eel, *Anguilla anguilla* (Linnaeus, 1758) [32]. In addition, *E. aquitanicus* coexists with several introduced fish species including the invasive pumpkinseed *Lepomis gibbosus* (Linnaeus, 1758) and the Eastern mosquito fish *Gambusia holbrooki* Girard, 1859. It also occurs in sympatry with the Northern pike *Esox lucius* Linnaeus, 1758, a species native to most of France but historically introduced into the southwestern region. *Esox lucius* is now widespread throughout France due to extensive introductions since the second half of the 20th century, often originating from central European hatcheries (Poland, Czechia, and Hungary) [9, 15, 23, 32, 37]. As a consequence, hybrids between the two pike species now occur within the distribution range of the Aquitanian pike [15, 17].

All these species in *E. aquitanicus* habitats may share parasites, allowing them to complete their life cycle. Non-native species such as the Northern pike or the pumpkinseed can introduce parasites that are not naturally present in the habitat through a phenomenon known as parasite spillover [12]. The presence of *Anguillicola crassus* Kuwahara, Niimi & Itagaki, 1974 in France illustrates a case of a non-native parasite being introduced to an endemic species: this parasite was introduced accidentally into Europe with the Japanese eel *Anguilla japonica* Temminck & Schlegel, 1846, in the 1980s [34]. This parasite threatens the European eel, which is currently classified as critically endangered [60].

Aside from the numerous environmental threats the Aquitanian pike shares with most other endemic species [10], such as the impacts of human activities, it is also threatened by hybridization and introgression with the Northern pike [15, 17, 32]. For these reasons, *E. aquitanicus* is considered a threatened species with a Vulnerable status on the French IUCN Red List [60]. Studying the parasitofauna of a threatened species can help to better understand potential environmental threats, such as overexploitation, habitat loss and fragmentation, effects of non-native species, and climate change [22].

To better understand the role of this fish in its natural habitat and its interactions with other species, we investigated the parasitic species that use the Aquitanian pike as a host. Pike parasites have been well studied across North America [18, 26, 55, 63], Eastern Europe [41, 52, 61, 64], and in Iran [51]. In nature, pikes are either definitive or intermediate hosts of various parasitic species, especially helminths (Cestoda, Nematoda, Trematoda). In the group of Cestoda for example, the Northern pike can be infected by *Triaenophorus* spp. in the intestine [7, 51, 61] and serves as an intermediate host for *Dibothriocephalus latus* (Linnaeus, 1758) Lühe, 1899, which infects its muscles and gut [21, 24].

Over a period of three years, Aquitanian pike were sampled to conduct a parasitological survey. Organs were collected in the field and subsequently analyzed in the laboratory. Recovered cestodes were photographed and measured. For molecular identification, we focused on compatible available sequences, targeting the ribosomal markers 18S and 28S as well as the mitochondrial marker, cytochrome oxidase subunit I (COI) [3, 36, 62]. The 28S marker, widely used in platyhelminths to resolve phylogenetic relationships, is relatively well conserved and allows comparison with a broad set of reference sequences. While 28S provides a solid framework for genus identification, COI offers higher resolution for distinguishing closely related species due to its high variability [44]. In this study, we used 28S and COI to identify the cestode species recovered from the Aquitanian pike.

Here, we report the first evidence of *Bothriocestus claviceps* (Goeze, 1782) in the intestine of the Aquitanian pike and pike hybrids, identified according to molecular data and general morphology*.*

## Material and methods

According to European legislation on animal welfare (Decree No. 2013-118 of February 1, 2013, Article R.214-89), the euthanasia of animals solely for the purpose of using their organs or tissues, when performed using approved methods, is not considered an experimental procedure. Consequently, no approval from an ethics committee was required for this study. Fish were euthanized according to established protocols. In 2021 and 2022, individuals were euthanized in water containing 10% clove oil, with death confirmed by the cessation of opercular movements and the absence of response to tactile stimuli [13]. In 2024, euthanasia was performed in two steps: initial anesthesia in a solution containing 150 mg/L MS-222 (tricaine methanesulfonate, fish anesthetic) and 150 mg/L NaHCO_3_, followed by a second bath containing 350 mg/L MS-222 and 150 mg/L NaHCO_3_ to ensure painless death.

A total of 64 pike were included in this study. Sampling was conducted during three electrofishing campaigns during the month of June in 2021, 2022, and 2024 in the Landes region (southwestern France), as part of fish-rescue operations aimed at transferring individuals from drying seasonal ponds to nearby rivers, in accordance with Prefectural Decree No. 2021-231, 2022-068, 2024-560. These operations, carried out by the Fédération Départementale des Landes pour la Pêche et la Protection des Milieux Aquatiques (FDAAPPMA40), primarily targeted the endemic Aquitanian pike, although other fish species such as the European eel and pumpkinseed were also present. The main study site was a duck hunting pond near Lit-et-Mixe (44.052100, –1.266470), which connects in winter to the “Courant mort” brook and serves as a breeding site for adult Aquitanian pike. This site was previously identified as hosting a pure population of *E. aquitanicus* without hybridization with *E. lucius* [14].

In 2021, 30 *E. aquitanicus* were selected from 1,907 fish captured at this site. In 2022, 7 *E. aquitanicus* were retained from 2,093 fish captured at the same location. In 2024, 27 pike were selected from 773 captured individuals. During this year, the sampling campaign included the Lit-et-Mixe site as well as additional locations known to host hybrids: the Ceyrolles channel in Bias (44.133333, −1.254166), Camp du Poteau in Retjons (44.184726, –0.276861), and Baratnaou brook in Solférino (44.133363, –0.978375). Of the 27 pike sampled, 21 were *E. aquitanicus* (2 from Lit-et-Mixe, 13 from the Ceyrolles channel, 3 from Baratnaou brook, and 3 from Camp du Poteau), 5 were hybrids (all from the Ceyrolles channel), and one pike from this site could not be identified.

Species identification in 2021 and 2022 was based on morphological criteria [17, 30], whereas in 2024 it was confirmed using molecular analysis following Denys *et al.*, (2023) [16] given the potential presence of hybrids at the new sites.

All fish in each campaign were measured, weighed, and tagged for individual identification. All procedures complied with national regulations and institutional guidelines for animal care and use. A parasitological assessment was performed on all selected specimens. External parasites were first examined on the skin and fins. The visceral cavity and adjacent musculature of each fish was thoroughly examined for the presence of parasitic cysts. Subsequently, the fins, gills, gonads, kidneys, and digestive tract were removed. To preserve parasite localization and to preserve parasite DNA, tissues were fixed in 80% ethanol. Samples were transported to the laboratory a few days later under Nagoya Protocol compliance (ABSCH-IRCC-FR-262903-1). Parasites were removed from the tissues and preserved in 90% ethanol. Here, we will only discuss cestodes, as the results concerning other parasites are still being analyzed.

Of the 64 pike specimens examined, 9 were parasitized with a total of 32 cestodes with scolex, 2 cestodes without scolex, and 1 larval cestode (Table 1). Fragments of strobila without scolex were also observed but were not counted as specimens, except when fragments of cestodes were found in a pike without scolex during examination, meaning that the scolex had been missed or lost. All specimens were photographed (Leica M205 C using a K3C camera (Leica Microsystems GmbH, Wetzlar, Germany)), except the larval cestode, which was fully used for DNA extraction before imaging. After imaging, we measured total length, maximum width, mature segment length and width, scolex length, terminal disc width, and neck width using Leica Application Suite Software. Depending on their size, either entire specimens or portions of cestodes were preserved for molecular analysis. Specimens, particularly those fragmented, were used for molecular identification. Morphometric measurements were additionally performed to provide supplementary descriptive data on the cestode species. The preserved specimens were incorporated into the collection of the Museum national d’Histoire naturelle (MNHN) under the collection number MNHN-130YT.

**Table 1:** List of parasitized pikes and cestodes identified during parasitological examination over the three years of the study.

Pike tag number	Year of collection	Sampling location	Host species	Host total length (mm)	Parasite	Parasite location	Number of cestodes found
19588	2021	Lit-et-Mixe (France)	*Esox aquitanicus*	165	Cestode larvae	Anterior intestine	1
19597	2022	Lit-et-Mixe (France)	*Esox aquitanicus*	196	Mature cestode without scolex	Anterior intestine	1
18104	2022	Lit-et-Mixe (France)	*Esox aquitanicus*	231	Piece of cestode	Posterior intestine	1
18108	2022	Lit-et-Mixe (France)	*Esox aquitanicus*	172	Pieces of cestode with scolex	Intestine	14
M17	2024	Lit-et-Mixe (France)	*Esox aquitanicus*	296	Piece of cestode	Anterior intestine	1
M5	2024	Ceyrolles channel (France)	*Esox aquitanicus*	136	Pieces of cestode with scolex	Posterior intestine	7
M24	2024	Ceyrolles channel (France)	*Esox aquitanicus*	155	Entire cestode	Anterior intestine	1
303	2024	Camp du Poteau (France)	*Esox aquitanicus*	670	Piece of cestode with scolex	Anterior intestine	1
M23	2024	Ceyrolles channel (France)	*Esox aquitanicus × Esox lucius*	183	Entire cestode	Anterior intestine	1
					Pieces of cestode with scolex	Anterior intestine	4

Parasite DNA was extracted using a QIAamp^®^ DNA Micro Kit (QIAGEN, Hilden, Germany), following the manufacturer’s instructions, and eluted in 50 μL to maximize DNA yield.

In 2021, following the first detection of cestode larvae in Aquitanian pike, a preliminary molecular identification was performed using the 18S marker amplified with primers 18E 5′ CTG GTT GAT CCT GCC AGT 3′ and 18R 5′ CTA CGG AAA CCT TGT TAC G 3′ [25]. This marker was used only for the initial identification of the larval specimen, as subsequent amplification attempts targeting 28S and COI were unsuccessful for this sample. DNA was amplified by PCR in a final volume of 20 μL containing 5% DMSO, 1 μL BSA at 1 mg mL^−1^, 1 μL of dNTP 6.6 mM, 0.15 μL of QIAGEN Taq DNA polymerase, using 2 μL of the buffer provided by the manufacturer, 0.4 μL of each primer at 10 μM, and 2 μL of DNA extract. PCR amplification was carried out on a t100^TM^ thermal cycler (Bio-Rad Laboratories, Inc., Hercules, CA, USA), using the following cycling conditions: 5 min at 94 °C, followed by 40 cycles of 30 s at 94 °C, 30 s at 54 °C, and 1 min at 72 °C, with a final elongation of 7 min at 72 °C. PCR products were visualized on ethidium bromide-stained agarose gels, and positive amplicons were sequenced by the Sanger method by Eurofins (http://www.eurofins.fr) using the same primers.

For all other cestode specimens, molecular identification was performed using the partial 28S marker with primers LSU-5 5′ TAG GTC GAC CCG CTG AAY TTA AGC 3′ and 1500R 5′ GCT ATC CTG AGG GAA ACT TCG 3′ [43]. DNA was amplified by PCR in a final volume of 20 μL, using the same reaction components and concentrations as described above for 18S. After 3 min denaturation at 94 °C, PCR was run for 45 cycles (1 min at 94 °C; 1 min at 50 °C; 2 min at 72 °C), followed by a 10-minute terminal elongation at 72 °C on a Bio-Rad T100™ thermal cycler. Successful PCRs were selected on ethidium bromide-stained agarose gels. Sequencing was performed on an Illumina^®^ MiSeq platform at the Concarneau marine station, Muséum national d’Histoire naturelle (MNHN), using a MiSeq reagent kit 250 V2 (Illumina, Inc., San Diego, CA, USA) to generate paired-end reads. The partial 28S sequences were quality-checked and assembled with Geneious Prime^®^ 2024.0.5 software (www.geneious.com).

COI amplification was subsequently attempted to refine species-level identification but repeatedly failed using standard PCR protocols. To overcome this limitation, shotgun sequencing was performed on selected specimens with sufficient DNA quantity, as measured using a Qubit 2.0 Fluorometer (Thermo Fisher Scientific, Waltham, MA, USA). Paired-end sequencing was carried out on an Illumina NextSeq 2000 at the iGenSeq platform (ICM, Paris, France). COI and additional 28S sequences were recovered from shotgun reads in Geneious Prime^®^ 2025.0.5 using a reference-mapping approach with *B. claviceps* sequences available in GenBank (accession numbers KR780818 for COI and KR780910 for 28S, with lengths of 600 and 1,941 bp, respectively). Sequencing depth averaged 139× for COI (range: 26–327.4×) and 283.54× for 28S (range: 199.3–328.6×). In addition to individually processed specimens, one shotgun library was generated from a pooled sample containing DNA from three cestodes in equimolar concentrations. Following sequencing and reference mapping, no sequence variation was detected among reads originating from this pool for either the COI or 28S markers. As the three specimens were morphologically similar and yielded identical consensus sequences, three identical sequences were retained and treated as separate individuals in subsequent analyses. Detailed shotgun sequencing statistics are provided in Supplementary Table 1. All GenBank accession numbers obtained in this study and the lengths of the corresponding sequences are listed in Table 2.

**Table 2:** Freshwater Bothriocephalidae taxa and the two outgroups included in the phylogenetic analysis, along with newly generated sequences from this study, including host information and GenBank accession numbers. Each row corresponds to a single cestode specimen.

Species	Family	Environment	Host species	Tag number	COI GenBank Accession Numbers	28S GenBank Accession Numbers	References
*Bothriocestus claviceps* (Goeze, 1782)	Bothriocephalidae	Freshwater	*Esox aquitanicus* Denys, Dettai, Persat, Hautecoeur & Keith, 2014	19597	–	PV491892** 1,473 bp	This study
*Bothriocestus claviceps*	Bothriocephalidae	Freshwater	*Esox aquitanicus*	M5T8	PZ796962* 600 pb	PZ798912* 1,491 bp	This study
*Bothriocestus claviceps*	Bothriocephalidae	Freshwater	*Esox aquitanicus*	M5T9	PZ796961* 600 pb	PZ798911* 1,491 bp	This study
*Bothriocestus claviceps*	Bothriocephalidae	Freshwater	*Esox aquitanicus*	M5T11	PZ796960* 600 pb	PZ798910* 1,491 bp	This study
*Bothriocestus claviceps*	Bothriocephalidae	Freshwater	*Esox aquitanicus*	M5T4	PZ796963* 600 pb	PV491888** 1,473 bp	This study
*Bothriocestus claviceps*	Bothriocephalidae	Freshwater	*Esox aquitanicus*	M5T2	PZ796964* 600 pb	PV419889** 1,478 bp	This study
*Bothriocestus claviceps*	Bothriocephalidae	Freshwater	*Esox aquitanicus*	M24T1	PZ796957* 600 pb	PV419890** 1,473 bp	This study
*Bothriocestus claviceps*	Bothriocephalidae	Freshwater	*Esox aquitanicus × Esox lucius*	M23T4	PZ796958* 600 pb	PZ798908* 1,491 bp	This study
*Bothriocestus claviceps*	Bothriocephalidae	Freshwater	*Esox aquitanicus × Esox lucius*	M23T3	–	PV491891** 1,473 bp	This study
*Bothriocestus claviceps*	Bothriocephalidae	Freshwater	*Esox aquitanicus × Esox lucius*	M23T1	PZ796959* 600 pb	PZ798909* 1,491 bp	This study
*Bothriocestus claviceps*	Bothriocephalidae	Freshwater	*Esox aquitanicus*	303 T1	PZ796965* 600 pb	PV491893** 1,473 bp	This study
*Bothriocestus claviceps*	Bothriocephalidae	Freshwater	*Lepomis gibbosus* (Linnaeus, 1758)	–	MZ532576 548 bp	–	Kvach *et al.*, 2021
*Bothriocestus claviceps*	Bothriocephalidae	Freshwater	*Lepomis gibbosus*	–	–	MZ519069 1,004 bp	Kvach *et al.*, 2021
*Bothriocestus claviceps*	Bothriocephalidae	Freshwater	*Lepomis gibbosus*	–	MZ532577 551 bp	–	Kvach *et al.*, 2021
*Bothriocestus claviceps*	Bothriocephalidae	Freshwater	*Lepomis gibbosus*	–	–	MZ519070 984 bp	Kvach *et al.*, 2021
*Bothriocestus claviceps*	Bothriocephalidae	Freshwater	*Lepomis gibbosus*	–	MZ532578 551 bp	–	Kvach *et al.*, 2021
*Bothriocestus claviceps*	Bothriocephalidae	Freshwater	*Lepomis gibbosus*	–	MZ532579 551 bp	–	Kvach *et al.*, 2021
*Bothriocestus claviceps*	Bothriocephalidae	Freshwater	*Trinectes maculatus* (Bloch & Schneider, 1801)	–	KR780818 600 bp	KR780910 1,491 bp	Brabec *et al.*, 2015
*Bothriocephalus australis* Kuchta, Scholz & Justine, 2009	Bothriocephalidae	Coastal / Benthic	*Platycephalus aurimaculatus* Knapp, 1987	–	–	KR780886 1,523 bp	Brabec *et al.*, 2015
*Bothriocephalus scorpii* (Müller, 1776)	Bothriocephalidae	Coastal / Benthic	*Myoxocephalus scorpius* (Linnaeus, 1758)	–	KR780788 600 bp	AF286942 4,140 bp	Brabec *et al.*, 2015
*Bothriocephalus timii* Gil de Pertierra, Arredondo & Incorvaia, 2015	Bothriocephalidae	Coastal / Benthic	*Cottoperca gobio* (Günther, 1861)	–	KR780787 600 bp	KR780885 1,493 bp	Brabec *et al.*, 2015
*Bothriocephalus travassosi* Tubangui, 1938	Bothriocephalidae	Freshwater / Coastal	*Anguilla marmorata* Quoy & Gaimard, 1824	–	KR780820 600 bp	KR780912 1,545 bp	Brabec *et al.*, 2015
*Bothriocestus cuspidatus* (Cooper, 1917)	Bothriocephalidae	Freshwater	*Sander vitreus* (Mitchill, 1818)	–	KR780816 600 bp	KR780908 1,489 bp	Brabec *et al.*, 2015
*Bothriocestus kupermani* (Choudhury, Scholz & Beuchel, 2022)	Bothriocephalidae	Freshwater	*Lepomis gibbosus*	–	KR780814 600 bp	KR780906 1,489 bp	Brabec *et al.*, 2015
*Bothriocephalus* sp.	Bothriocephalidae	Freshwater	*Micropterus dolomieu* Lacepède, 1802	–	KR780813 600 bp	KR780905 1,489 bp	Brabec *et al.*, 2015
*Bothriocephalus* sp.	Bothriocephalidae	Freshwater	*Lepisosteus oculatus* Winchell, 1864	–	KR780815 600 bp	KR780907 1,489 bp	Brabec *et al.*, 2015
*Tetracampos ciliotheca* Wedl, 1861	Bothriocephalidae	Freshwater	*Clarias gariepinus* (Burchell, 1822)	–	KR780795 600 bp	JQ811835 1,502 bp	Brabec *et al.*, 2015
*Ichthybothrium ichthybori* Khalil, 1971	Bothriocephalidae	Freshwater	*Ichthyborus besse* (Joannis, 1835)	–	KR780793 600 bp	JQ811837 1,484 bp	Brabec *et al.*, 2015
*Schyzocotyle nayarensis* (Malhotra, 1983)	Bothriocephalidae	Freshwater	*Barilius* sp.	–	KR780829 582 bp	KR780922.1 1,583 bp	Brabec *et al.*, 2015
*Senga lucknowensis* Johri, 1956	Bothriocephalidae	Freshwater	*Mastacembelus armatus* (Lacepède, 1800)	–	–	KR780891 1,510 bp	Brabec *et al.*, 2015
*Senga magna* (Zmeev, 1936)	Bothriocephalidae	Freshwater	*Siniperca chuatsi* (Basilewsky, 1855)	*–*	KR780821 600 bp	KR780913 1,510 bp	Brabec *et al.*, 2015

*Sequences obtained using the shotgun sequencing approach. **Sequences obtained by PCR.

A total of 11 28S sequences and 9 COI sequences were obtained in this study (Table 2).

To support the molecular identification of the cestodes recovered in this study, two phylogenetic analyses were performed. Taxon selection was based on the phylogeny of freshwater bothriocephalids proposed by Brabec *et al.* (2015) [3]. Sequences were aligned using MAFFT [31] implemented in Geneious Prime^®^ 2025.0.5, which was also used to calculate *p*-distances. The best-fitting evolutionary models were selected using JModelTest 2.1.1 [11] under the Bayesian Information Criterion (BIC). The 28S dataset consisted of 25 taxa and an alignment length of 1,581 bp and was analyzed under the GTR + G model. For the COI analysis, taxa represented by short sequences were excluded when their respective clades remained represented by other species, resulting in a dataset of 25 taxa and an alignment length of 547 bp. The GTR + I + G model was selected for the COI dataset.

Phylogenetic trees were reconstructed using Bayesian inferences in MrBayes v. 3.2.6 [49]. Two independent analyses were run for 5 million generations, sampling every 200 generations. The convergence of the two analyses was assessed using TRACER v. 1.6.0 [47]. Majority-rule consensus trees were generated after discarding the first 25% of sampled trees as burn-in and were visualized using FigTree v. 1.4.0 [46]. Three sequences belonging to the H clade defined by Brabec *et al.* (2015) [3] were included as outgroups in the 28S analysis: *Bothriocephalus scorpii* (GenBank: AF286942), *Bothriocephalus timii* (GenBank: KR780885), and *Bothriocephalus australis* (GenBank: KR780886), and only 2 were used for the COI analysis due to the small size of *B. australis* (*Bothriocephalus scorpii*, GenBank: KR780788; and *Bothriocephalus timii,* GenBank: KR780787).

To describe the parasitic infection parameters, three standard parasitological indices were calculated following the definitions provided by Bush *et al.* (1997) [5]. Intensity was defined as the number of individuals of a given parasite species per infected host. Abundance corresponded to the number of individuals of a given parasite species per examined host. Prevalence was expressed as the percentage of infected hosts among all examined hosts. These indices were calculated for each year of sampling, for each sampling location, and for each host species (*E. aquitanicus* or hybrids with *E. lucius*).

## Results

### Parasitological indices

Parasitological indices varied across sampling years and host species (Table 3a). In 2021, *E. aquitanicus* showed a low parasite prevalence of 3%, with only one individual infected out of a total of 30 examined, carrying a single larval cestode (intensity = 1; abundance = 0.03). In 2022, the prevalence was found to be markedly higher (43%), with parasite loads ranging from 1 to 14 cestodes per host (intensity = 5.3; abundance = 2.3). During the 2024 campaign, 4 out of 21 *E. aquitanicus* were found to be infected (prevalence = 19%), with parasite numbers varying from 1 to 7 (intensity = 2.5; abundance = 0.5). Additionally, among hybrids sampled in 2024, 1 out of 5 individuals were infected with 5 cestodes (prevalence = 20%; intensity = 5; abundance = 1).

**Table 3:** List of a) different parasitological indices calculated annually for each host species, and b) parasitological indices of *Esox aquitanicus* and hybrids by sampling location for the 2024 campaign. Prevalence is expressed as a percentage. Dashes indicate that no parasites were detected at the site.

a)	Year	Host Species	Intensity	Abundance	Abundance Range	Prevalence	Total Host Infected
	2021	*E. aquitanicus*	1	0.03	1	3	1 on 30
	2022	*E. aquitanicus*	5.3	2.3	1–14	43	3 on 7
	2024	*E. aquitanicus*	2.5	0.5	1–7	19	4 on 21
	2024	*E. aquitanicus × E. lucius*	5	1	5	20	1 on 5
b)		Sampling location	Intensity	Abundance	Abundance Range	Prevalence	Total Host Infected
		Camp du Poteau	1	0.3	1	33	1 on 3
		Baratnaou brook	–	–	–	–	0 on 3
		Lit-et-Mixe	1	0.5	1	50	1 on 2
		Ceyrolles channel	4	0.6	1–7	15	2 on 13

In 2024, parasitological indices were evaluated across the four sampling locations (Table 3b). In Retjons, 1 out of 3 examined individuals was infected (prevalence = 33%), with a single cestode observed (intensity = 1; abundance = 0.3). In Lit-et-Mixe, 1 out of 2 fish was infected (prevalence = 50%) and carried one parasite (intensity = 1; abundance = 0.5). At the Ceyrolles channel, 2 of 13 sampled individuals were infected (prevalence = 15%), with parasite loads ranging from 1 to 7 (intensity = 4; abundance = 0.6). No cestode infections were recorded at Baratnaou brook.

### Morphology

A total of 32 cestodes were collected (Table 1), representing different developmental stages ranging from larvae to gravid adults. In *E. aquitanicus* from Lit-et-Mixe, six specimens were non-gravid and six were gravid adults. The developmental stage of the cestodes recovered from Ceyrolles channel could not be determined, as only the anterior part was found (i.e., scolex). Among the cestodes recovered from hybrid pike, only one complete non-gravid specimen was recovered. All morphometric data are summarized in Table 4. The largest cestode recovered (from Aquitanian pike tagged 19597 in Lit-et-Mixe) was estimated to be approximately 67 mm long. Because the specimen was incomplete and partially damaged during dissection and handling, its total length could not be measured accurately. The larval cestode collected in 2021 could not be measured due to the absence of photographic documentation.

**Table 4:** Morphological and morphometric parameters of *Bothriocestus claviceps* from *Esox aquitanicus*, with comparative data from previous studies.

Locality	Lit-et-Mixe, France		Ceyrolles channel, France			Camps du Poteau, France	Gryfino Canal, Poland	Brodowski Pond, Poland	Europe	Canada
Reference	This study		This study	This study		This study	Kvach *et al.,* 2021	Kvach *et al.,* 2021	Scholz 1989	Scholz 1997
Host	*Esox aquitanicus*		*Esox aquitanicus*	*Esox aquitanicus* × *Esox lucius*		*E. aquitanicus*	*Lepomis gibbosus*	*Lepomis gibbosus*	*Anguilla anguilla*	*Anguilla rostrata*
Maturity stage	Non-gravid	Adult	Undetermined*	Non-gravid	Undetermined*	Undetermined*	Non gravid	Adults	Adults	Adults
Total length (mm)	1.6–3.2 (2.4)	3.5–13.7 (7.2)	/	3.3		/	4.8	19.8–31.2	400	24–133
Maximum width	167–258 (220)	222–588 (416)	/	199		/	0.6	0.9–1	3.2	0.87–4.95
Mature segment (L)	63–222 (125.6)	78–350 (185)	/	131		/	331–385	335–852	/	120–1,080
Mature segment (W)	167–258 (230)	222–588 (416)	/	191		/	498–559	344–722	/	711–440
Length/width ratio	0.31–1.39 (0.75)	0.22–0.75 (0.56)	/	0.7		/	1:15–1:1.45	1:3.6–4.6	/	1:2.3–5.6
Scolex (L)	507–744 (615)	501–859 (653)	350–573 (467)	427	428	/	/	636–733	1300 – 2,100	240 – 1,140
Scolex (W)	194–286 (240)	192–259 (221)	138–194 (169)	107	181	/	/	224–229	440–880	224–650
Terminal disc (W)	267–336 (289)	224–332 (266)	130–251 (188)	169	136–185 (165)	194	/	201–202		189–472
Neck (W)	145–205 (171)	132–230 (168)	76–330 (139)	69	134	/	/	113–122	240–320	112–128
Number of cestodes measured**	6	6	8	1	3	1				

Measurements are given in μm, except for total length (mm). L: length; W: width; () brackets indicate mean values; * Sexual maturity not determined; only the scolex was recovered; **Not all cestodes could be fully measured.

### Molecular analysis

Across the three years of sampling, 12 cestodes from 6 pikes were successfully identified by molecular analysis: 1 cestode in 2021, 1 in 2023, and 10 in 2024 (from 4 different hosts).

PCR amplification targeting the 28S marker had a success rate of 43% (6 out of 14 samples). Shotgun sequencing succeeded for all processed samples and yielded five additional 28S sequences.

In 2021, an initial molecular identification was carried out using the 18S marker, and BLAST analysis of the resulting 816 bp sequence (GenBank: PZ798907) led to the presumptive identification of *B. claviceps* (100% identity and 0 gaps with KR780957.1 from Brabec *et al.* 2015 [3]). However, given the limited taxonomic resolution of the 18S marker, the sequence could only be confidently assigned to the genus *Bothriocestus*. For the following sampling years, 11 partial 28S sequences and 9 COI sequences were generated and used for phylogenetic analysis.

**Figure 1:**
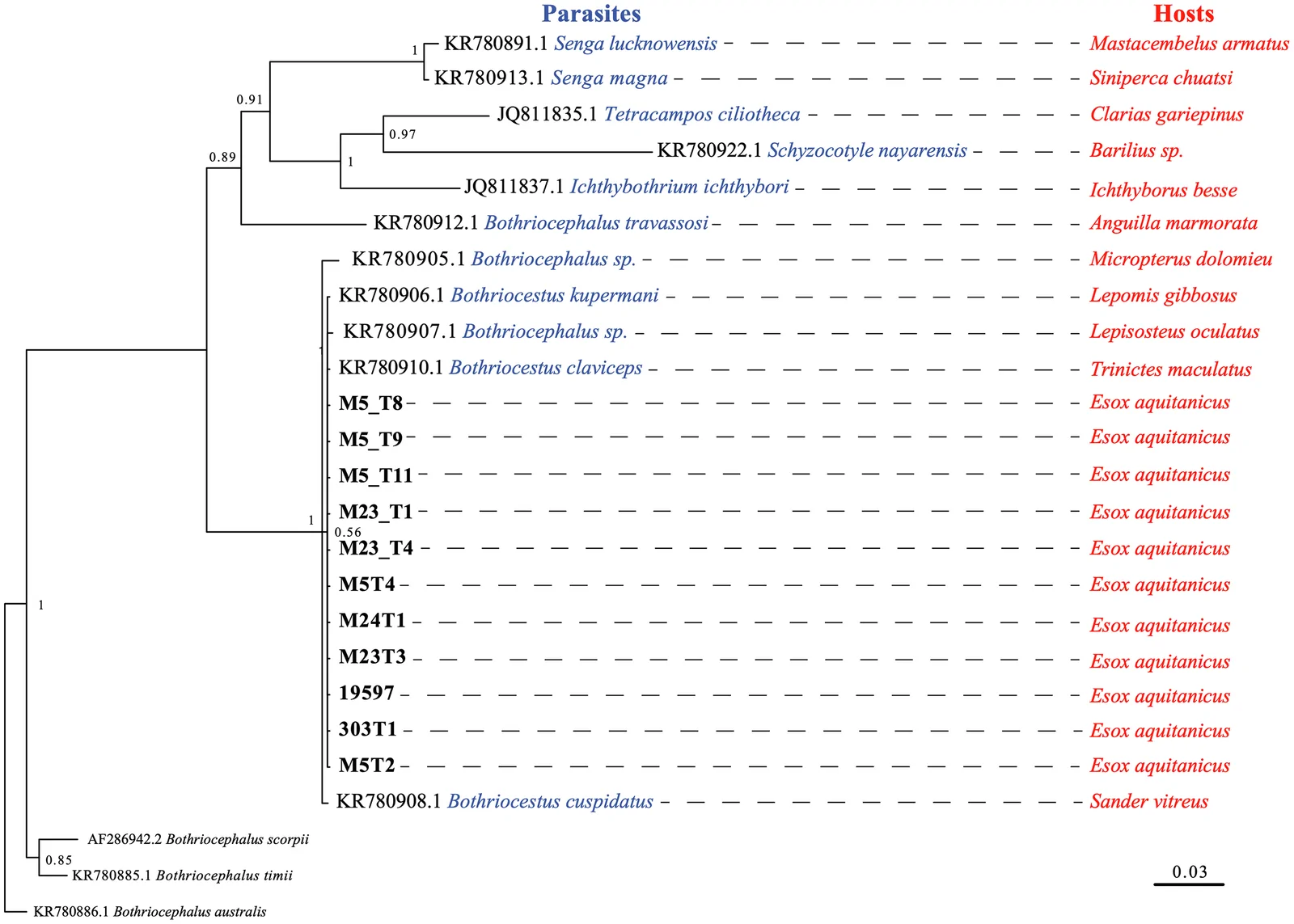
Phylogenetic tree by Bayesian inference with the partial 28S rDNA marker (1,581 bp) on 25 sequences of bothriocephalids including those from this study (in bold). Posterior probability values are indicated above the nodes. Outgroups are represented by *Bothriocephalus scorpii* (AF286942), *Bothriocephalus timii* (KR780885), and *Bothriocephalus australis* (KR780886).

A phylogenetic tree for the partial 28S rDNA marker (1,581 bp) by Bayesian inference from 25 sequences was performed including bothriocephalids (Table 2). Our 11 sequences grouped well with strong robustness with one sequence of *B. claviceps*, one sequence of *Bothriocestus cuspidatus* (Cooper, 1917), and one of *Bothriocestus kupermani* Choudhury, Scholz & Beuchel, 2022 (Fig. 1). The 28S marker grouped well the *Bothriocestus* genus but did not enable discrimination at the species level with 0.18% genetic divergence between *B. claviceps* and *B. kupermani*, and 0.28% between *B. claviceps* and *B. cuspidatus*. However, our sequences were 100% identical to the *B. claviceps* reference sequence.

**Figure 2:**
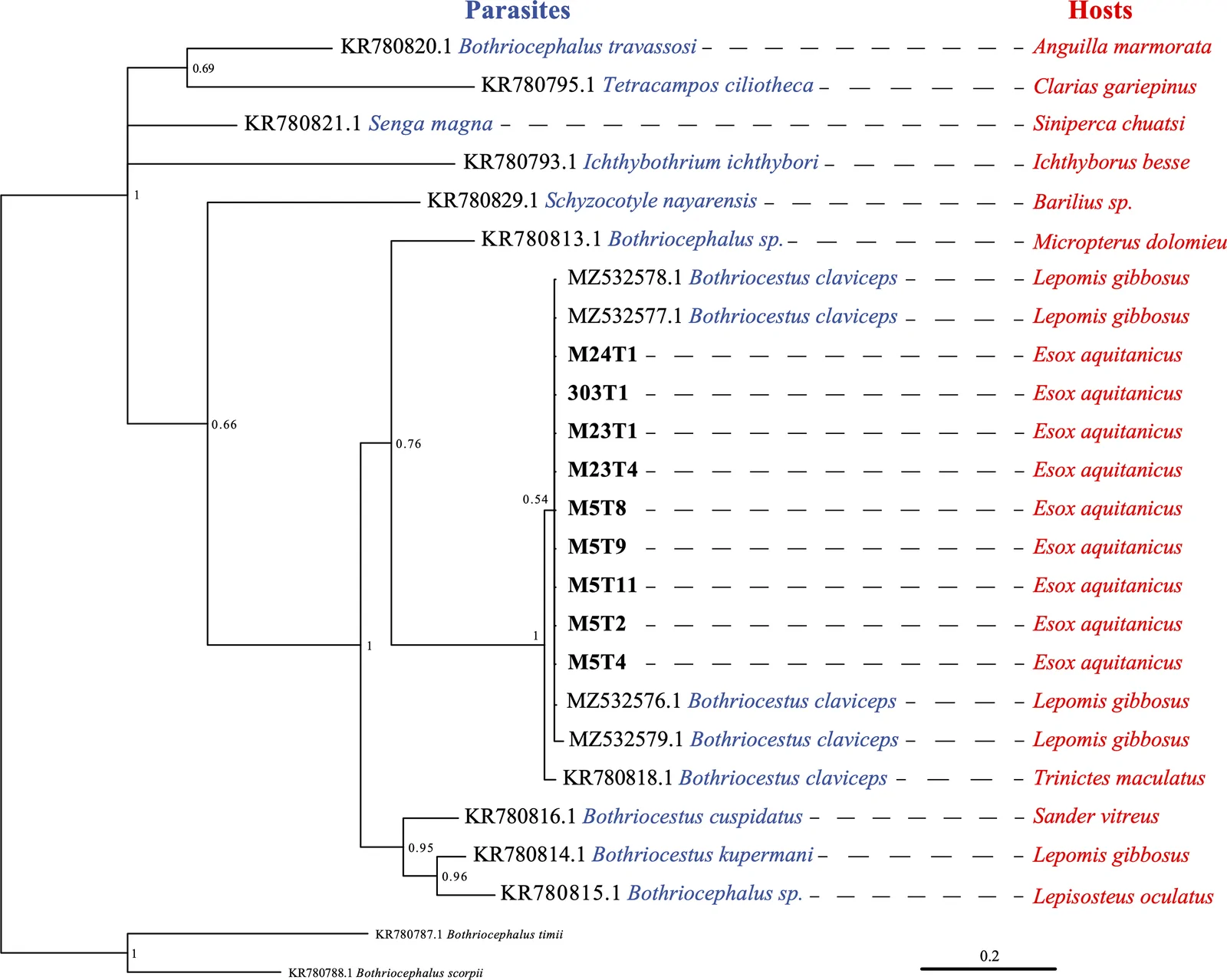
Phylogenetic tree by Bayesian inference with the CO1 marker (547 bp) on 25 sequences of Bothriocephalids including those from this study (in bold). Posterior probability values are indicated above the nodes. Outgroups are represented by *Bothriocephalus scorpii* (KR780788) and *Bothriocephalus timii* (KR780787).

To elucidate the relationships among these three species, an additional phylogenetic tree based on the COI marker (547 bp) by Bayesian inference from 25 sequences was performed (Fig. 2). Our sequences clustered within the *B. claviceps* clade, which was clearly separated from *B. kupermani* and *B. cuspidatus*. The genetic divergence between the *B. claviceps* clade and these two species reached 12.46% and 12.54%, respectively.

In addition, two sequences identified in GenBank as *Bothriocephalus* sp. clustered within the *Bothriocestus* clade in the 28S and COI tree, together with *B. claviceps*, *B. cuspidatus*, and *B. kupermani*.

## Discussion

Bothriocephalid tapeworms are common parasites that infect teleost fishes in marine, brackish, and freshwater environments [3, 6, 48, 55]. However, no Bothriocephalid has been reported in pike (*Esox* spp*.*), except for one accidental infection by *Bothriocestus cuspidatus*, after the fish consumed prey carrying the parasite [57]. For the first time and across three different years, we report the presence of *B. claviceps* cestodes from this family in the coastal river basins of the Landes department, with an overall prevalence of 14% over the three-year period in *E. aquitanicus* and in one of five hybrids of *E. aquitanicus* with *E. lucius* (Table 3). During the first year of sampling, only a single larval specimen was recovered, whereas in the following two years, non-gravid and gravid specimens were observed. In comparison, the prevalences observed in our study are higher than those reported by Kvach et *al.* (2021) [36] in the paratenic host pumpkinseed, where only two adult cestodes were recorded (prevalence of 5% and 10%, in different places). In the European eel, prevalence appears to vary seasonally (0 to 40%), with a peak in May to June [19, 42]. Overall, the prevalences observed in our study were lower than the mean prevalence of 21% calculated from the data compiled by Jakob *et al.,* (2016) [28] for the European eel. However, prevalence values reported in the studies included in this review showed considerable variation, likely reflecting differences in ecological characteristics among study sites.

*Bothriocestus claviceps* was detected in two distinct coastal drainages: the Courant de Contis (Lit-et-Mixe) and the Courant de Mimizan drainages (including the three additional sites from 2024). Parasitological indices varied among sampling sites; however, these differences should be interpreted with caution given the low number of pike examined at certain locations (3 in Camp du Poteau, 2 in Lit-et-Mixe, and 3 at Baratnaou brook) (Table 3). It is also worth noting that the method used to collect and preserve the intestinal content for molecular analyses was not optimal for recovering cestodes, as it tented to damage and fragment the parasites. Beyond sampling efforts and methods, environmental factors may also contribute to spatial variation [2] including water temperature, the availability of the intermediate host, and the seasonal variation in the occurrence and maturation of cestodes [8]. Although the sites share similar characteristics, such as oligotrophic conditions, sandy substrates, acidic and clear waters, and vegetation typical of the Landes department, differences in zooplankton and fish community composition may occur. In particular, the parasite depends on trophic transmission through an intermediate host copepod such as *Macrocyclops albidus* (Jurine, 1820) [29, 56] and may also involve paratenic fish hosts such as *Perca fluviatilis* Linnaeus, 1758 before reaching its definitive host [29, 40, 53, 56]. Therefore, the occurrence and distribution of *B. claviceps* may be influenced by the local availability and distribution of both intermediate and paratenic hosts.

**Figure 3:**
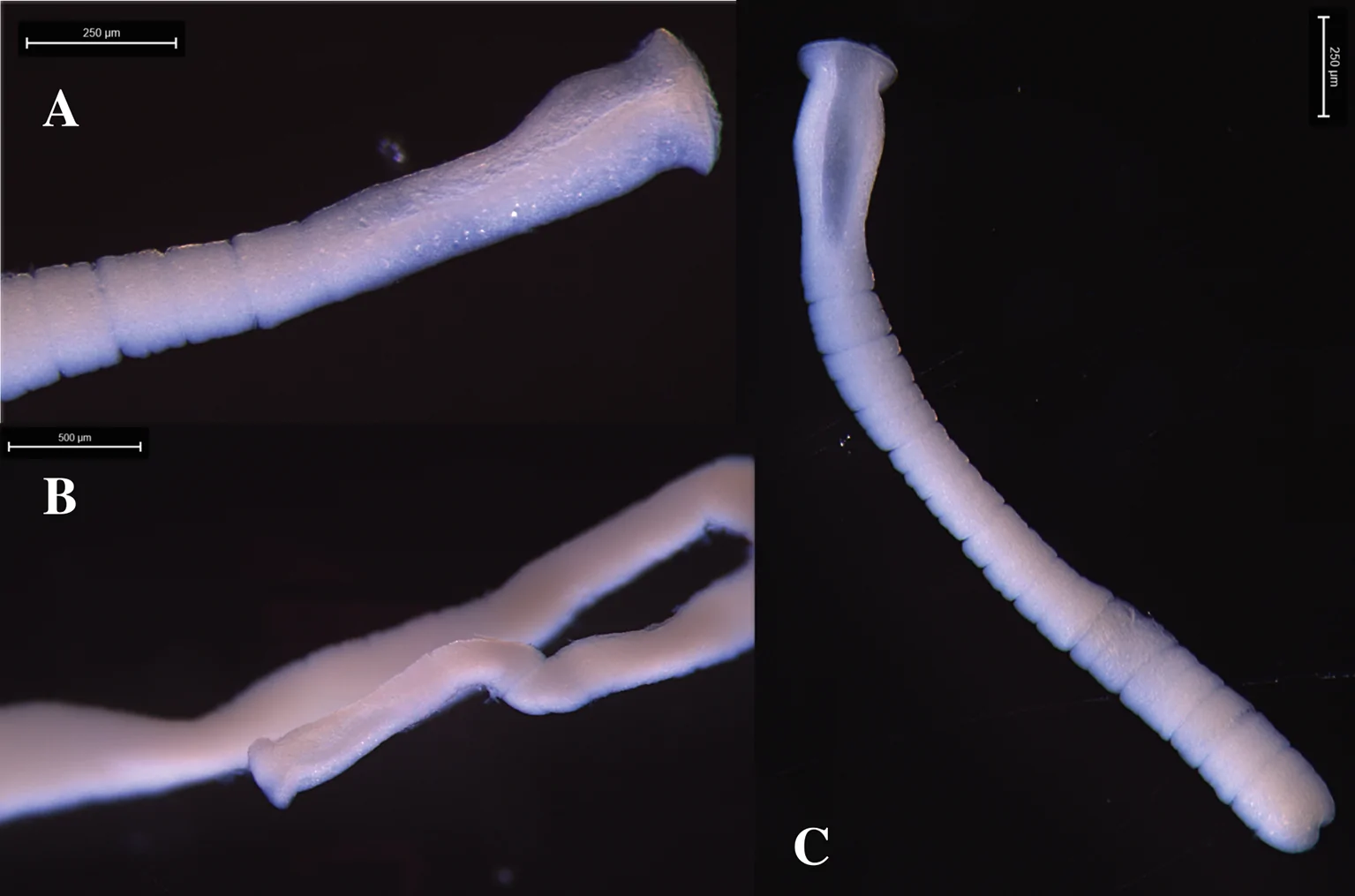
*Bothriocestus claviceps* from *Esox aquitanicus* photographed using a Leica M205C stereomicroscope driven by LASX software. A) 18108-T7, B) 18108-T9, and C) 18108-T8 from the 2022 sampling campaign. Cestodes specimens have been deposited in the collections of the Muséum national d’Histoire naturelle (MNHN, Paris, France) under collection lot number MNHN-130YT.

Regarding our general observations on morphology, we noted that the scolex was elongated and narrow in all specimens examined. The anterior ends appeared convex, rounded and distinctly bilobed, with two longitudinal grooves clearly visible along its length (Fig. 3). These characteristics are consistent with morphological features of the Bothriocephalidae family, including the genus *Bothriocestus* [57]. The specimens also had a prominent terminal disc wider than the scolex itself, corresponding to the description of *B. claviceps* by Scholz (1997) (Fig. 3) [55]. Our non-gravid specimens were on average approximately half the size of non-gravid cestodes reported by Kvach *et al.,* (2021) [36]. Mature individuals with gravid proglottids were also smaller than those documented in the literature [36, 54, 55]. In contrast, scolex length was generally comparable to published values, except in the *B. claviceps* recovered from *A. anguilla*, in which the scolex reached up to 2.1 mm. Also, the scolex lengths observed in our study fall within the range reported for *B. claviceps* in *Anguilla rostrata* (Lesueur, 1817), although in this host, the *B. claviceps* scolex was up to 1,140 μm in length, whereas the lengths in specimens from our study did not exceed 744 μm (Table 4). Overall, our cestodes appeared to be smaller than those previously reported for *B. claviceps*. However, species-level identification in the present study was not based primarily on morphology, as specimens were collected and preserved in 80% ethanol for molecular analyses, limiting the observation of some diagnostic morphological characters. Scholz (1997) [55] emphasized the occurrence of numerous misidentifications of *B. claviceps*. Considering these documented difficulties and our collection method, our study relied on molecular data, while morphological observations were used solely to supplement existing data on this species and for general observation.

All sequences obtained in this study clustered within a well-supported *Bothriocestus* clade together with reference sequences available in GenBank. However, as previously reported, the 28S marker provided limited resolution at the species level and did not allow a clear distinction among *B. claviceps*, *B. kupermani*, and *B. cuspidatus* (Fig. 1). To further assess the taxonomic identity of our specimens, an additional Bayesian phylogenetic analysis based on COI marker was performed (Fig. 2). The analysis based on the COI marker recovered a well-supported *Bothriocestus* clade. All sequences obtained in the present study clustered within the *B. claviceps* lineage together with previously published reference sequences from GenBank, including specimens recovered from *L*. *gibbosus* and *Trinectes maculatus* (Bloch & Schneider, 1801). None of our sequences grouped with *B. kupermani* or *B. cuspidatus*, which formed distinct lineages within the genus. These results support the identification of all specimens collected from *E*. *aquitanicus* and hybrid as *B*. *claviceps*.

*Bothriocestus claviceps* is a cestode with Holarctic distribution, found across North America, Europe, North Africa, and introduced in Japan [1, 33, 35, 40, 42, 55, 59]. *Anguilla anguilla* and *A. rostrata* have long been recognized as its main definitive hosts [29, 56]. Other species, such as the hogchoker (*Trinectes maculatus*), and the smooth newt (*Lissotriton vulgaris* (Linnaeus, 1758)), have also been documented as potential definitive hosts [3, 58]. Additional fish species, such as the European perch *P. fluviatilis, G. holbrooki*, and *L. gibbosus*, may be involved in the life cycle as paratenic hosts [20, 36, 40]. The pumpkinseed *L. gibbosus* may act as both a definitive and a paratenic host [36]. However, our molecular and morphological data suggest that the Aquitanian pike and its hybrids may represent suitable hosts for *B. claviceps*. Of the 32 cestodes recovered, seven (22%) exhibited clear signs of strobilation and contained gravid segments. Although the occurrence of gravid cestodes may, in some cases, result from postcyclic transmission following the ingestion of an infected definitive host, our observations are also consistent with successful development and maturation of the parasite within Aquitanian pike and their hybrids. The repeated detection of the parasite over three sampling years, including both immature and gravid specimens, argues against an isolated accidental infection event and suggests that *B. claviceps* is regularly transmitted to the Aquitanian pike within these coastal ecosystems.

**Figure 4:**
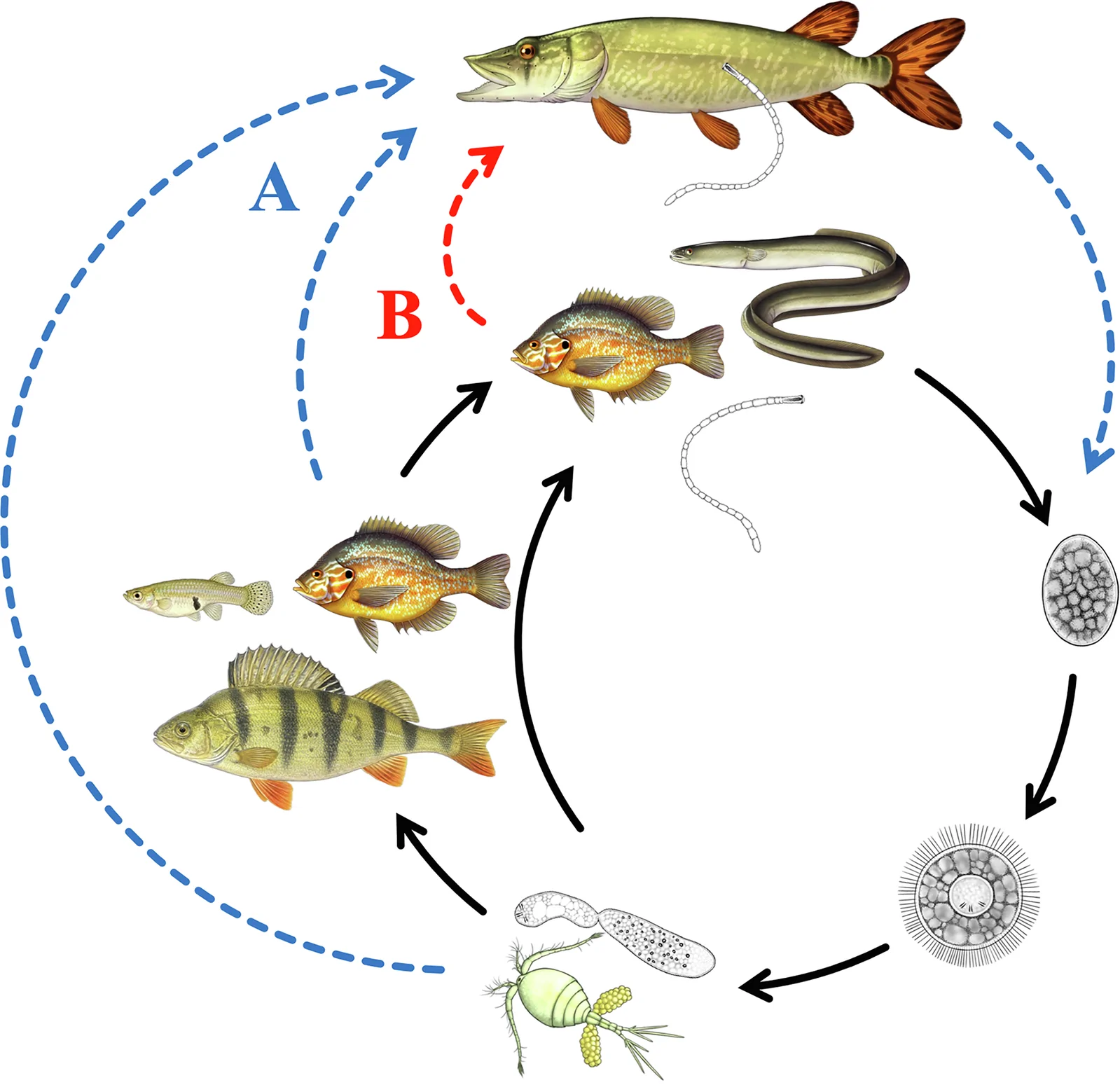
Life cycle of *Bothriocestus claviceps*, a cestode parasite of eels. Black arrows indicate transmission pathways documented in the literature. The life cycle involves a copepod intermediate host, followed by paratenic hosts such as the European perch (*Perca fluviatilis*), pumpkinseed (*Lepomis gibbosus*), and mosquitofish (*Gambusia holbrooki*). The principal definitive host is the European eel (*Anguilla anguilla*), although the pumpkinseed has also been suggested as a potential definitive host. (A) Hypothetical scenario in which the Aquitanian pike (*Esox aquitanicus*) acts as a definitive host, allowing the completion of the parasite’s development (blue arrows). (B) Alternative scenario in which the Aquitanian pike acts as a postcyclic host following the consumption of infected hosts (red arrows).

As a carnivorous predator, *E. aquitanicus* may acquire *B. claviceps* through several transmission routes (Fig. 4). During its early life stages, juvenile pikes are primarily microphagous and feed on zooplankton, including copepods [4]. At this stage, infection may occur through the ingestion of infected intermediate hosts carrying a larva. If pike are suitable definitive hosts, these larvae may subsequently develop and mature within the intestine. As pike grow, they undergo an ontogenetic dietary shift towards piscivory, typically within their first year of life [27, 38]. Larger individuals increasingly prey upon fish, including conspecifics, and may therefore acquire the parasite through the consumption of infected paratenic or potentially definitive hosts. Consequently, juvenile cestodes recovered from pike may originate either from direct transmission through infected copepods or from the ingestion of infected paratenic hosts (Fig. 4A). Gravid worms may result from the successful development of larvae acquired earlier through copepods or paratenic hosts. However, an alternative explanation must also be considered. A well-documented example involves the Northern pike *E*. *lucius*, which may acquire the cestode *Proteocephalus percae* (Müller, 1780) through the consumption of infected European perch *P. fluviatilis*, even though the latter represents the obligate definitive host of the parasite [39]. Similarly, if the Aquitanian pike act as postcyclic hosts, mature *B. claviceps* could be acquired through the consumption of infected definitive hosts and subsequently persist within the digestive tract (Fig. 4B). Distinguishing between these scenarios is therefore essential when assessing the role of pike in the life cycle of *B*. *claviceps*.

Among the fish species potentially involved in the transmission of *B. claviceps*, Dupont and Gabrion (1986) [20] experimentally demonstrated that the mosquitofish *G. holbrooki* and the pumpkinseed *L. gibbosus*, two North American species introduced into France, can serve as paratenic hosts. More recently, Kvach *et al.* (2021) [36] reported *L. gibbosus* as a paratenic host and suggested that it may additionally act as a definitive host. In our study area, both introduced species are abundant in Lit-et-Mixe and Camp du Poteau, whereas the Ceyrolles channel additionally harbors *P. fluviatilis*, another paratenic host implicated in the life cycle of *B. claviceps*. No information is currently available for the fish community of Baratnaou brook, although the presence of pumpkinseed and mosquitofish is considered likely given their widespread distribution in the area. Based on these fish communities, several trophic pathways may facilitate the transmission of *B. claviceps* to *E. aquitanicus* (Fig. 4). While infection initially requires the ingestion of an infected copepod, transmission to pike may subsequently occur through the consumption of infected paratenic or definitive fish hosts. Pumpkinseed, mosquitofish, and, in the Ceyrolles channel, European perch therefore represent plausible routes of transmission. European eel (*A. anguilla*), the main definitive host of *B. claviceps*, was also present at all study sites and may contribute to the maintenance and transmission of the parasite. However, eels may represent a less frequent source of infection in our study system, as they are generally consumed less frequently by pike than the fish species mentioned above, particularly by the relatively small individuals examined in this study. This is consistent with our stomach content observations, which revealed numerous fish scales and otoliths, as well as undigested pumpkinseeds and mosquitofish, whereas no eel remains were observed.

Given the limited number of specimens examined and the condition of some of the recovered cestodes, additional studies would be required to determine more precisely the role of Aquitanian pike and their hybrids in the life cycle of *B. claviceps.* Improved collection protocols specifically designed to preserve intact specimens, combined with detailed morphological examinations using permanent whole-mount preparations, would allow a more accurate assessment of cestode maturity and reproductive status. In addition, experimental infection trials, similar to those conducted by Dupont and Gabrion (1986) [19], would provide the most direct evidence for determining whether *B. claviceps* can complete its development and reproduce within these hosts. Future studies based on larger sample sizes could help clarify the role of Aquitanian pike in the life cycle of *B. claviceps*. In addition to experimental infections, ecological approaches similar to those developed by Dupont and Gabrion (1987) [20] with the eel, could be used to investigate relationships among host size and infection parameters.

Although *B. claviceps* has never been reported in the Northern pike, its presence in *E. aquitanicus* and hybrids raises the question of whether *E. lucius* could also serve as a possible host in this region. As part of this study, we examined the intestinal parasitic fauna of two *E. lucius* individuals from the same watershed and found no cestodes in these fish. However, these specimens were line-caught from a large, anthropized water body, distinct from the seasonal ponds and small streams where the Aquitanian pikes were sampled. Given that the infection rate of *B. claviceps* in *E. aquitanicus* was relatively low (one in five individuals infected in 2024), the absence of the parasite in these two *E. lucius* specimens may simply reflect chance and insufficient sampling. Further investigations using improved sampling methods and larger sample sizes of *E. lucius* from a wider range of habitats are needed to clarify the potential role of the Northern pike in the life cycle of *B. claviceps* in this region. In addition, examining parasite communities in potential paratenic hosts such as *L. gibbosus*, *G. holbrooki*, and *P. fluviatilis* where they co-occur with *E. aquitanicus* would help assess their contribution to parasite transmission. Screening local populations of *A. anguilla* for the presence of *B. claviceps* would also provide valuable information, although such investigations may be constrained by the conservation status of the species. Together, these approaches would help clarify the transmission pathways of *B. claviceps* and the respective roles of intermediate, paratenic, definitive, and postcyclic hosts within these ecosystems.

## Conclusions

This study provides the first record of *B. claviceps* in the Aquitanian pike *E. aquitanicus* and in one hybrid with *E. lucius*. The parasite was detected repeatedly over three sampling years and in multiple coastal river basins of the Landes department, demonstrating that *B. claviceps* is established within these ecosystems. Molecular analyses based on 28S and COI sequences consistently identified all recovered specimens as *B. claviceps* and confirmed their affiliation with previously published reference sequences. The recovery of both juvenile and mature cestodes, including gravid individuals, indicates that Aquitanian pike can participate in the life cycle of *B. claviceps*. However, the precise role of *E. aquitanicus* and its hybrids remains unresolved, and the available data do not allow us to determine whether these fish function as definitive hosts, postcyclic hosts, or both. Further studies combining improved morphological preservation, experimental infections, and broader ecological surveys will be necessary to clarify the transmission pathways of *B. claviceps* and the contribution of pike to its life cycle.

## Data Availability

All sequences used in this article were deposited in GenBank (https://www.ncbi.nlm.nih.gov/genbank/) with the following accession numbers: PV491888, PV491889, PV491890, PV491891, PV491892, PV491893, PZ798907, PZ798908, PZ798909, PZ798910, PZ798911, PZ798912, PZ796957, PZ796958, PZ796959, PZ796960, PZ796961, PZ796962, PZ796963, PZ796964, PZ796965. The authors used Artificial Intelligence (AI) to assist with English language editing and grammatical corrections; all scientific content and interpretations remain the responsibility of the authors. AI was also used to generate the illustrations of *Perca fluviatilis* and *Gambusia holbrooki* in Figure 4. The manuscript was subsequently reviewed and corrected by a native English speaker (USA).

## Supplementary material

*Supplementary Table 1*: Coverage statistics of the sequences obtained by shotgun sequencing, for the mitochondrial COI and nuclear 28S markers. The supplementary material of this article is available at https://www.parasite-journal.org/10.1051/parasite/2026046/olm.
