# Supplementary material for: Bothriocestus claviceps (Cestoda: Bothriocephalidae) in pike in southwestern France (Teleostei: Esocidae): molecular evidence
Source: Parasite. 2026 Aug 26;33:46. doi: 10.1051/parasite/2026046 (PMC13521508; doi:10.1051/parasite/2026046)
Supplement: Supplementary file 1 [file parasite-33-46-s1.pdf]

Supplementary Data 1: Coverage statistics of the sequences obtained by shotgun sequencing, for the mitochondrial COI and nuclear 28S markers. Mean, minimum, and maximum coverage depth (in reads), standard deviation (Std Dev), percentage of the reference sequence covered (Ref Seq, %), and number of mapped reads (Nb of reads) are indicated for each sample.

	Sample	Mean	Minimum	Maximum	Std Dev	Ref Seq	Nb of reads
	303T1	148	109	181	18,4	100	1115
	M23T1	92,7	69	129	13,7	100	776
	M23T4	26	13	40	5,1	100	317
	M24T1	65,7	48	90	9,1	100	514
**COI**	M5T8	157,4	128	194	14	100	1390
	M5T9	157,4	128	194	14	100	1390
	M5T11	157,4	128	194	14	100	1390
	M5T2	119,5	95	138	8,4	100	808
	M5T4	327,4	266	398	26,6	100	2730
	M5T8	328,6	272	44	28,1	100	6111
	M5T9	328,6	272	44	28,1	100	6111
**28S**	M5T11	328,6	272	44	28,1	100	6111
	M23T4	232,6	169	282	26,2	100	4643
	M23T1	199,3	160	252	17,2	100	3726
